# Influence of funding fads and donor interests on international aid for conservation in Madagascar

**DOI:** 10.1111/cobi.70122

**Published:** 2025-08-15

**Authors:** Johanna Eklund, Marketta Vuola, Satu Määttänen, Katia Nakamura, Jeremy Brooks, Daniel C. Miller

**Affiliations:** ^1^ Department of Geosciences and Geography, Faculty of Science University of Helsinki Helsinki Finland; ^2^ Global Development Studies, Helsinki Institute of Sustainability Science, Faculty of Social Sciences University of Helsinki Helsinki Finland; ^3^ Viikki Tropical Resources Institute, Department of Forest Sciences, Faculty of Agriculture and Forestry University of Helsinki Helsinki Finland; ^4^ Keough School of Global Affairs University of Notre Name Notre Dame Indiana USA; ^5^ School of Environment and Natural Resources, College of Food, Agricultural, and Environmental Sciences Ohio State University Ohio USA

**Keywords:** conservation funding, conservation policy, international aid, nongovernmental organizations, protected areas, áreas protegidas, ayuda internacional, financiamiento para la conservación, organizaciones no gubernamentales, políticas de conservación

## Abstract

Tens of billions of dollars in official development assistance have been spent over the past three decades to address the increasingly rapid loss of biodiversity globally. Despite this expenditure, detailed knowledge of who has provided these funds and who has used them, for what purpose, where, why, and with what consequences remains limited. To address this gap, we used a mixed‐methods approach to map and analyze international aid for biodiversity conservation in Madagascar, a high‐priority country for conservation. We combined collation and analysis of publicly available funding data with semistructured interviews with a range of conservation actors in Madagascar. Overall, biodiversity aid to the country declined from 1990 to 2018 and was punctuated by sharp declines during times of political unrest. Funding flows were marked by periods with distinctive emphases, from institutional development to protected areas, to creating market‐based incentives for conservation. These patterns reflected key donor interests and resonated with the views and perceptions of conservation practitioners on the ground. Conservation professionals highlighted how administrative shortsightedness and imbalances in the power relations shaping conservation aid allocation have led to an increasing projectification of the conservation sector and weakening of state capacity. Our findings show that by studying how funding for biodiversity changes within countries over time, one can reveal the interests and power dynamics among donors, governments, and nongovernmental organizations that influence funding decisions and conservation efforts. The evidence and insights presented here can inform future biodiversity funding decision‐making in Madagascar and elsewhere and have particular relevance given major funding commitments under the Kunming–Montreal Global Biodiversity Framework.

## Abstract (es)

Influencia de las modas de financiamiento y los intereses de los donantes sobre la ayuda internacional para la conservación en Madagascar

**Resumen:**

En las últimas tres décadas se han gastado decenas de miles de millones de dólares en ayuda oficial al desarrollo para hacer frente a la pérdida cada vez más rápida de la biodiversidad a nivel mundial. A pesar de este gasto, todavía es poco el conocimiento detallado sobre quién ha proporcionado estos fondos y quién los ha utilizado, con qué fin, dónde, por qué y con qué consecuencias. Para subsanar este vacío, utilizamos un enfoque de métodos mixtos para mapear y analizar la ayuda internacional destinada a la conservación de la biodiversidad en Madagascar, un país de gran prioridad para la conservación. Combinamos la recopilación y el análisis de datos de financiamiento disponibles públicamente con entrevistas semiestructuradas a diversos actores de la conservación en Madagascar. En general, la ayuda a la biodiversidad en el país disminuyó entre 1990 y 2018 y se vio interrumpida por fuertes descensos durante los periodos de inestabilidad política. Los flujos de financiación se caracterizaron por periodos con énfasis distintivos, desde el desarrollo institucional hasta las áreas protegidas, pasando por la creación de incentivos basados en el mercado para la conservación. Estos patrones reflejaron los intereses clave de los donantes y coincidían con las opiniones y percepciones de los profesionales de la conservación sobre el terreno. Los profesionales de la conservación destacaron cómo la miopía administrativa y los desequilibrios en las relaciones de poder que configuran la asignación de la ayuda a la conservación han llevado a una creciente «proyectivización» del sector de la conservación y al debilitamiento de la capacidad del Estado. Nuestros hallazgos muestran que, al estudiar cómo cambia el financiamiento de la biodiversidad dentro de los países a lo largo del tiempo, se pueden revelar los intereses y las dinámicas de poder entre los donantes, los gobiernos y las ONG que influyen en las decisiones de financiación y los esfuerzos de conservación. Las pruebas y los conocimientos aquí presentados pueden servir de base para la toma de decisiones futuras sobre la financiación de la biodiversidad en Madagascar y en otros lugares, y tienen especial relevancia dados los importantes compromisos de financiamiento contraídos en el marco del Marco Mundial para la Diversidad Biológica de Kunming‐Montreal.

## INTRODUCTION

Biodiversity loss and ecosystem collapse are among the greatest threats facing humanity (Díaz et al., 2019; World Economic Forum, 2024). Yet, the biodiversity crisis has remained underrecognized and underfunded (Deutz et al., 2020; IPBES, 2019). Much of global biodiversity occurs in economically poor countries (Grenyer et al., 2006), which are heavily aid reliant (Deutz et al., 2020; Miller et al., 2013; Waldron et al., 2013). Yet, surprisingly little knowledge exists on the funding landscape for biodiversity conservation, especially within countries. Global (Miller et al., 2013; Waldron et al., 2013) and regional studies exist (Qin et al., 2022) but few present detailed quantitative analyses at country level (cf. Devkota et al., 2023; Salcido et al., 2009). Such country‐level analyses combined with insights from interviews and other qualitative data are crucial for understanding how the wider aid sector interacts with national policy developments, local stakeholders, and practical conservation work, and ultimately how conservation funding might be made more effective.

Extensive reliance on foreign aid is criticized for trapping low‐income countries in dependencies in a system in which aid often benefits donors more than the recipients (Fox, 2020; Gibson et al., 2005; Moss et al., 2006; Olivié & Pérez, 2019; Pankaj, 2005) A large proportion of aid accrues to donor countries themselves (e.g., through disbursements to implementing nongovernmental organizations (NGOs) and private contractors based in those countries or contracts for vehicles, computers, and other equipment derived from the donor country), which can dramatically reduce the amount of funding to recipient countries and limit aid effectiveness (Easterly & Pfutze, 2008; Sims, 2023). Aid is also used to exert influence in recipient countries (Alesina & Dollar, 2000; Browne, 2012), including conservation policies. For example, the United States has sought to brand illegal wildlife trade as a national security interest domestically and in aid recipient countries, thereby justifying further military and other expenditures (Massé & Margulies, 2020). At the same time, increased taxpayer demand in donor countries for transparency in aid expenditures has led to greater emphasis on monitoring and evaluation as donors seek to demonstrate accountability and effectiveness (Elbers & Arts, 2011).

These aid dynamics have influenced conservation by promoting short‐term projects often implemented by NGOs rather than national governments (Sayer & Wells, 2004). This projectification of public policies (Sjöblom, 2009) has affected the role of national governments in conservation by simultaneously moving responsibilities up (toward supranational institutions), down (to regional and local levels), and out (toward the private sector and semipublic institutions) (Pierre & Peters, 2000). These shifts have often sidelined the central state, weakening institutions and diminishing long‐term support (Barrett et al., 2001; Dietrich, 2021; Razafindrakoto et al., 2017). In this context, international conservation NGOs (Corson, 2010; Méral, 2012) and for‐profit development contractors based in donor countries (Nagaraj, 2015) have seen their influence grow, thereby exacerbating existing power asymmetries, which often have colonial roots (Collins et al., 2021). Together, these changes form part of a larger trend toward neoliberalism—the idea that the state should have only a minimal role in managing economic affairs, with market approaches to meet public needs favored overall (Biebricher, 2019)—and within conservation specifically (Buscher et al., 2012).

Madagascar presents an ideal context in which to explore these shifting dynamics in international conservation aid. The country's unique but highly threatened fauna and flora have made it a global biodiversity priority (Brooks et al., 2006). Its biodiversity importance together with its status as one of the world's poorest countries (World Bank, 2024) has meant Madagascar ranks as a top‐10 recipient of biodiversity aid (Miller et al., 2013), with some 84% of the country's total biodiversity funding originating from international aid (Waldron et al., 2013). Despite this financial support, the country's forest cover has decreased markedly over the past decades (Vieilledent et al., 2018), and Madagascar has the highest number of threatened species of any country (IUCN, 2019). These conditions have led many to conclude that conservation in the country has been ineffective (Corson, 2016; Freudenberger, 2010; World Bank, 2021). Although this may be true overall, research in which counterfactual methods were applied suggests that some approaches to conservation have been relatively effective and that without them the situation in Madagascar would be even more dire (Devenish et al., 2022; Eklund et al., 2016, 2019).

We sought to contribute to debates about international aid generally and conservation funding in Madagascar more specifically by presenting new evidence on biodiversity aid flows to the country. Our objectives were to track biodiversity aid allocated to Madagascar since 1990, describe the nature of this aid, analyze trends over time, compare biodiversity aid with the wider aid landscape in Madagascar, and draw out implications for future research and policy.

To realize these objectives, we drew from the multidisciplinary literature on conservation and development funding in Madagascar and beyond and employed a mixed‐methods approach. We systematically compiled and analyzed available data on biodiversity‐related aid flows in Madagascar and complemented this with interviews with in‐country local experts working in conservation. With this approach, we sought to provide a systematic, in‐depth understanding of the conservation funding landscape in Madagascar, the underlying factors driving funding decision‐making, and how funding dynamics shaped different conservation approaches over time. Our results take on particular relevance (Corson, 2016; Devkota et al., 2023; Waeber et al., 2016) given global commitments to significantly increase conservation funding to reach targets under the 2030 Kunming–Montreal Biodiversity Framework (CBD, 2022).

To help structure our analysis and aid interpretation of our findings, we briefly review the history of environmental policies in Madagascar over the past three decades. In January 1990, a National Environmental Action Plan (NEAP)—a kind of unofficial structural adjustment loan condition—took force in Madagascar with an unusually long 15‐ to 20‐year vision (Kull, 2014). Conservation efforts under this plan and beyond evolved over time, and different phases were marked by significant political and strategic shifts.

### Environmental Program 1 (1990–1996)

During this early phase, the government of Madagascar established key institutions, such as Office National pour l’Énvironnement (ONE), Association Nationale d'Actions Environmentales (ANAE), and Association Nationale pour la Gestion des Aires Protégées (ANGAP) (later Madagascar National Parks [MNP]), to manage environmental activities, soil management, and protected areas (PAs), respectively (Freudenberger, 2010; Kull, 2014). This period saw a shift in focus from sustainable natural resource management broadly to a more specific emphasis on biodiversity, largely influenced by major funders, such as the U.S. Agency for International Development (USAID) and the World Bank (Corson, 2016). Community‐based resource management gained traction toward the end of this period, reflecting global conservation trends (Agrawal & Gibson, 1999). The 1996 GELOSE legislation (from Gestion Locale Sécurisée) sought to empower local communities with some resource management responsibilities (Pollini et al., 2014).

### Environmental Program 2 (1997–2002)

Amid political upheaval and the impeachment of President Zafy, followed by the re‐election of former dictator Didier Ratsiraka, evaluations of the first program were not favorable and the next phase transitioned to a regional approach where international funders focused on different parts of the country (Kull, 2014; Moreau, 2008). The period saw debates over the cumbersome nature of GELOSE legislation, with the United States supporting development of an alternative model, *Gestion Contractualisée des Forêts* (GCF), which simplified forest management without requiring tenure allocation or municipal negotiations (Kull, 2014).

### Environmental Program 3 (2003–2009)

Political instability continued with contested presidential elections and protests. President Ravalomanana announced a significant expansion of PAs at the 2003 World Parks Congress (branded the Durban vision), aiming to triple the PA network within 5 years (Corson, 2014). This expansion, however, faced criticism from stakeholders concerned about its impact on local communities (Kull, 2014). In 2005, the SAPM policy (*Système des Aires Protégées de Madagascar*) was introduced, centralizing PA authority under the Ministry of Environment and Forests but distributing management responsibilities across various actors, including MNP and conservation NGOs (Gardner et al., 2018). The establishment of the Madagascar Biodiversity Fund in 2005 supported these initiatives. During Ravalomanana's presidency, conservation strategies shifted toward a more neoliberal approach, emphasizing self‐sustainability through financial mechanisms (Kull, 2014). This led to the implementation of projects, such as Reducing Emissions from Deforestation and Forest Degradation (REDD+) and payment for ecosystem services (PES), across the country (Brimont & Bidaud, 2014).

### Post‐NEAP period (Post–2009)

The NEAP ended in 2009 with another political crisis. Ravalomanana was forced into exile, and Andry Rajoelina took power in what Western countries called a coup d'état. In response, most of the major donors (e.g., the United States, the World Bank, the European Union, and the African Development Bank) withheld nonhumanitarian funding to Madagascar (Ramiarantsoa et al., 2014). A democratic election was held in 2013, with Hery Rajaonarimampianina (ally to Rajoelina) winning. Rajoelina subsequently won elections twice (in 2018 and 2023). Little systematic research is available on donor responses to these political vicissitudes and the extent to which the neoliberalization of the conservation agenda has persisted since 2009.

## METHODS

We applied an interdisciplinary mixed‐methods approach (Kinnebrew et al., 2021). We combined quantitative and qualitative methods to examine the complex dynamics shaping conservation aid in Madagascar. Following Greene et al.’s (1989) typology, we employed this combination to strengthen the validity of our findings (triangulation) and expand knowledge by using different methods and data to address different aspects of our research questions (complementarity).

### Collation and categorization of funding data

To quantify aid flows, we sourced publicly available data on official development assistance (ODA) and other official flows (OOF) to Madagascar from Organization for Economic Co‐operation and Development (OECD) (2014–2018) and AidData (1990–2013). All information was compiled into a database for further processing and checked for duplicates to avoid double counting. Clearly faulty listings were removed, such as rows with missing amounts, amount given as a date, and one instance of a negative funding amount (resulting in 339 rows being removed from the full database of 15,463 entries). We focused on the years 1990–2018 to cover NEAP and post‐NEAP periods up to the time of our in‐country field research. Funding amounts were collected as commitments, not disbursements, given more limited data on the latter. To standardize amounts across time, we used deflators to convert nominal U.S. dollars to constant 2018 U.S. dollars (US$) (i.e., purchasing power of the base year 2018). This common approach to enabling comparable aid data works by adjusting for price (inflation) and changes in exchange rates (details in Appendix S1).

We categorized the cases (i.e., a funding commitment appearing in the funding database) as biodiversity‐related funding, non‐biodiversity‐related funding, or unclear or insufficient information (details in Appendix S2).

Categorization was carried out based on a combination of search words and filtering by Creditor Reporting System (CRS) sector codes (used by donors to classify their funding in a standardized way) and Rio markers (used by donors to denote their contributions under the Rio Convention) (OECD, 2024a). The most relevant sectors (details in Appendix S2) were screened manually by J.E. and S.M., and the whole database was screened using biodiversity conservation‐related keywords in 6 languages (Appendix S3) to further identify relevant funding flows. Cases for which it was difficult to determine the category were discussed between S.M. and J.E. until a consensus was reached. For cases with mixed conservation and development objectives, available data did not specify which proportion of the committed amount was directed to biodiversity, so we included the whole amount as biodiversity‐related aid, as has been done in previous studies (e.g., Miller, 2014).

### Further coding of the biodiversity related funding cases

Cases marked as 1 (biodiversity related) were further coded according to recipient, conservation actions, and whether the funding was related to PAs.

We categorized recipient type as Madagascar based, non‐Madagascar based, collaboration between Madagascar based and non‐Madagascar based, intergovernmental, or unknown (insufficient information). We also categorized recipients as comprising one of the following sectors: state, private, or NGO (at local, national, and international NGO levels and including parastatals, such as MNP, and Malagasy conservation associations), education, intergovernmental organizations, or a mix of these, plus unknown if information was insufficient.

We categorized the conservation actions according to the International Union for Conservation of Nature Conservation Actions Classification Scheme (Conservation Standards, 2019; Salafsky et al., 2008). To the scheme's 10 categories (Figure 5; Appendix S4), we added a category 11 (actions related to PAs, unspecified) for PA‐related actions for which it was impossible to determine whether the funding was for infrastructure building, planning, establishing a PA (category 6), or hiring staff or PA management (category 10).

For ease of interpretation, these categories were further grouped into 4 main action clusters. The management or restoration of sites or species cluster (categories 1–2) entailed actions related to land or species management with the underlying objective to reduce the direct stress on habitats or populations. The behavioral change (categories 3–5) cluster included actions aimed at awareness raising, law enforcement, or improving local livelihoods and creating economic incentives. The actions in the enabling conditions (categories 7–10) were related to law and policy frameworks, education, capacity building, and institutional development. Finally, support for PAs (categories 6 and 11) included actions related to area‐based conservation.

### Quantitative analyses

We charted overall funding trends from 1990 to 2018 and trends within the 4 phases of Madagascar's NEAP as presented above. Because NEAP periods differ in length, we report shares of conservation actions and patterns through time rather than absolute funding amounts. We used Excel 2019 for analyses and generating figures and R 4.5.0 for correlation analyses (Pearson).

### Qualitative data and coding

Qualitative data were derived from 15 in‐depth, semistructured interviews conducted with a range of conservation actors in 2019–2022 in Antananarivo, Madagascar, and via video calls (due to the COVID‐19 pandemic) (the interview protocol is in Appendix S9). Interviewees were selected to represent different Malagasy and international conservation organizations, including practitioners, funders, state institutions, and international consultants. Participants were originally identified based on personal networks (J.E., M.V., and D.C.M.), but we also sent interview requests to organizations and used snowballing techniques to ensure a diversity in respondents and organizations represented.

The interviews were transcribed and analyzed using qualitative content analysis (QCA) (Schreier, 2012). We used a 2‐staged approach to inductive coding, combining a concept‐driven and a data‐driven approach (Schreier, 2012). First, a round of coding was carried out to organize the data. This step offered a broad understanding of the content of the interview data and how it relates to previous studies and the data usefulness vis‐à‐vis the results of the quantitative analysis. The second round of coding was done with the specific purpose of investigating how the interviews might help explain our quantitative results.

In revision, OpenAI (the CurreChat building on GPT‐4) was used to shorten a previous longer version of the text on the background of conservation policy in Madagascar.

## RESULTS

### Changes in funding levels over time

**FIGURE 1:**
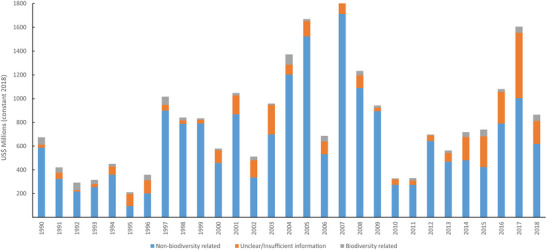
Trends in development aid (in constant 2018 US$) for Madagascar, 1990–2018 (gray, biodiversity‐related funding; blue, non‐biodiversity‐related funding; orange, funding that could not be classified as either).

**FIGURE 2:**
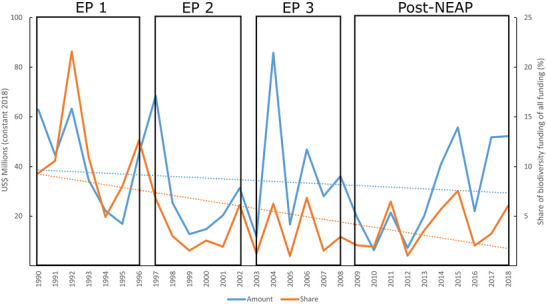
Biodiversity‐related funding (blue line, in millions of constant 2018 U.S. dollars) and share of total biodiversity funding (orange line) over time (dotted lines, linear time trend; rectangles, conservation policy periods described in “INTRODUCTION”; EP 1, 1990–1996 Environmental Program 1; EP 2, 1997–2002 Environmental Program 2; EP 3, 2003–2008 Environmental Program 3; Post‐NEAP, 2009–2018 post‐National Environmental Action Plan).

Madagascar received US$23,179 million in overall ODA from 1990 to 2018; the trend showed a slight increase (Pearson correlation coefficient = 0.38, *p* = 0.04) (Figure 1; Appendix S5). Biodiversity‐related funding comprised 4.25% (US$986 million) of this total, and the share of biodiversity‐related funding decreased over the years (Pearson correlation coefficient = −0.51, *p* = 0.0051) (Figure 2; Appendix S5). Non‐biodiversity‐related funding showed an increasing trend overall (Pearson correlation coefficient = 0.27, *p* = 0.15), whereas biodiversity‐related funding decreased (Pearson correlation coefficient = −0.14, *p* = 0.48), but these were not statistically significant (Figures 1 & 2; Appendix S5). Funding from the United States, the largest donor during the study period, declined significantly. When controlling for this decline, overall biodiversity aid from other donors showed a slight increase, although biodiversity aid still declined compared with overall ODA for Madagascar (Appendix S6), indicating the large role the United States has played in the conservation sector.

Each environmental program (EP) started with a pulse of funding (in 1992, 1997, and 2004). Funding peaked in 2004 after President Ravalomanana's 2003 promise to triple the country's PA network. This pledge garnered significant attention in the conservation sector and, according to one of our informants, gave confidence to funders, such as Germany and France, creating a momentum “like no other” (International consultant, 2019). In contrast, funding reached its all‐time low in 2010 after the political coup in December 2009. Democratic elections in 2013 seemed to stabilize the country in the eyes of donors, who again increased funding (Figure 2). The first EP saw the largest share of biodiversity‐related funding (5–22% of total ODA annually). Since then, this share has oscillated between 2% and 6% annually (Figure 2). The oscillations hint at a heavy reliance on project funding and the need to ensure continued funding after every 3‐ to 5‐year project cycle (Figure 2). Continuation of funding seemed linked to the political situation in the country, but informants also reported donors increasingly waiting to see how projects performed before deciding on continued funding.

### Funding entities and funded activities

**FIGURE 3:**
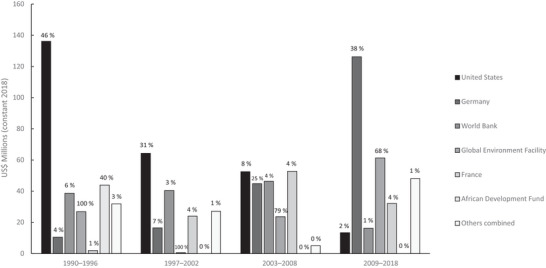
Changes in biodiversity‐related funding flows from the largest 6 donors over the 4 policy periods studied (percentages above bars, percentage of each donor's total aid funding to Madagascar that was biodiversity related during each period).

Together, the top 3 donors within our time frame, the United States, Germany, and the World Bank, provided more than 60% of all biodiversity‐related funding (Table 1). France, the previous colonial power in Madagascar, gave 11.2% of the biodiversity‐related funding, a share similar to their overall aid contribution (12.5%) (Appendix S7). By contrast, the United States and Germany directed a higher proportion of their ODA to biodiversity, 27% and 20%, respectively, for biodiversity aid compared with 8.5% and 4.2%, respectively, for overall ODA (Table 1; Appendix S7). However, the proportions from different donors changed over time. For example, the United States was by far the biggest donor in EP 1, but their participation decreased during EP 2 and 3, and especially after the political coup in 2009 (Figure 3). In contrast, Germany progressively took over as the largest biodiversity donor (Figure 3).

**TABLE 1:** Biodiversity‐related aid funders and amount of funding to Madagascar from 1990 to 2018.

Funder	Funding (US$ millions)	Share of total biodiversity funding (%)
United States	266.2179	27
Germany	198.1862	20
World Bank	141.8524	14
Global Environment Facility	112.3887	11
France	110.9087	11
African Development Fund	43.93065	4
Norway	20.93612	2
Switzerland	18.27652	2
Netherlands	14.37859	1
European Communities/European institutions	11.48076	1
International Fund for Agricultural Development	10.926	1
United Kingdom	9.152187	0.9
Sweden	8.397245	0.9
Japan	4.906444	0.5
United Nations Development Program	3.885704	0.4
Finland	2.874607	0.3
Belgium	2.374288	0.2
World Bank—Carbon Finance Unit	2.356671	0.2
Canada	1.763952	0.2
United Arab Emirates	0.351998	0.04
Monaco	0.078779	0.008
Italy	0.043163	0.004
Austria	0.014421	0.001
Total	985.6819	100

**FIGURE 4:**
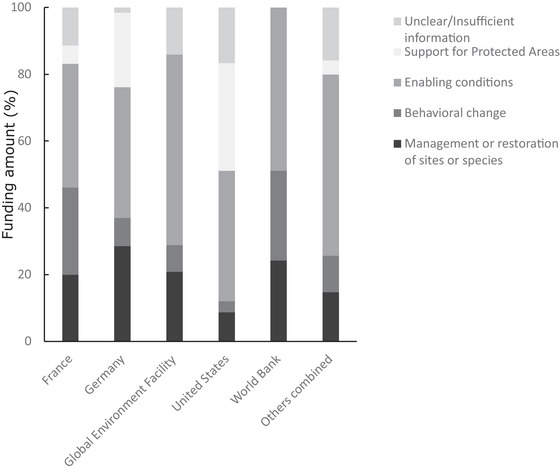
Top donor preferences for broad conservation actions as percentage of funding amount per conservation action.

Our thematic analyses of the data suggest clear preferences among donors to support particular conservation actions (Figure 4). All emphasized support for enabling conditions, such as developing conservation‐relevant institutions and policy. However, the United States had a strong focus on PAs, whereas France tended to support restoration and behavioral change projects. As the role of the United States diminished, Germany contributed a greater share of PA funding (Figure 3).

### Short termism and projectification in recent conservation funding

Interviewees highlighted short funding cycles (projects last about 1–4 years) (Figure 2) as especially challenging. They also noted that uncertainty about the timing of calls for proposals increased pressure to apply for as many opportunities as possible, regardless of their alignment with ongoing work or emerging needs. Proposal preparation diverted time and resources away from actual conservation work.

Short‐term project cycles were seen as especially challenging when working with and involving local communities in conservation. Several interviewees explained that it takes time and consistent effort to build trust in local communities. If funding ends after a few years, there is a serious risk of previous efforts being wasted, possibly even leaving local communities feeling resentful: “[W]e cannot stop the activities. It's our responsibility towards these people and towards the communities because… if the capacity is not yet there and you stop the activities all the previous effort will go to waste” (Malagasy NGO staff, 2019).

The projectification theme also emerged from interviews through funders’ recent reluctance to support ongoing management activities and running costs. For example: “At the beginning there was the Durban vision to triple the protected area of Madagascar. All funders wanted to fund only the creation of protected areas. Once the parks were created funders were not very willing to fund the management” (international nongovernmental organization [INGO] staff, 2019). Consequently, PA managers feel the need to keep searching for funding to cover management costs from several project baskets, even though actual project targets vary, depend on the donor, and are often very specific: “they don't want to finance the organization at all, only community activities” (Malagasy NGO staff, 2019). “Most difficult is to find funding for control and surveillance for the parks even though this should be our core activity. Donors want visibility: they are very eager to build a dam or a school where they can put their logo. You can't put your logo on surveillance” (INGO staff, 2019).

This projectification has created an environment in which conservation NGOs use their time adapting to the changing funding objectives to gather the funding to keep their team in the field. It explains why conservation practitioners start projects (e.g., tree planting) even if they are not convinced of their benefits. Interviews revealed that Malagasy NGOs find it especially difficult to maintain staff, follow their own vision, and ensure accountability to local communities. The two Madagascar‐based trust funds seem to be an exception to this trend because their funding focused on long‐term management, but only for a small group of selected PAs. Additional challenges identified were the lack of coordination and coherence (“We don't know if other NGOs are doing the same thing, funding a same activity in the same area” [Malagasy NGO staff, 2019].), which leads to competition and territoriality. “It is complicated. It is half the time working together and half the time competing against each other to get the same fund. This leads partly to distrust, and territoriality” (INGO staff, 2019). Further, different conditions set by donors along with heavy reporting requirements require specialized skills and capacities that smaller local actors do not have. These demands favor more well‐resourced international conservation NGOs. “The biggest part of funding is used in international NGOs and to hire foreign consultants” (Civil servant, 2019). “We have noticed that the donors don't have trust on national NGOs. When there is a competition, they choose the international ones” (Malagasy NGO staff, 2019).

### Funding fads and trends in conservation actions

**FIGURE 5:**
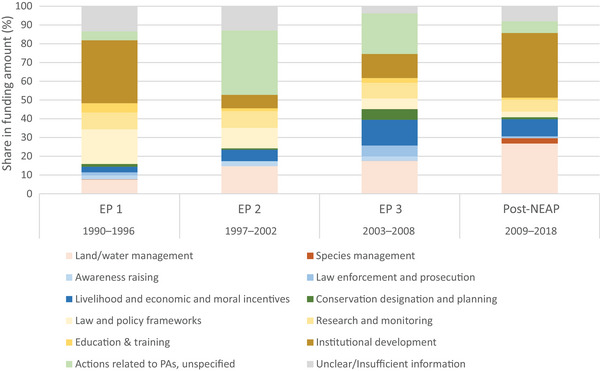
Conservation actions across all donors over the 4 policy periods inspected (EP, environmental program; NEAP, National Environmental Action Plan).

The most funded conservation actions were related to creating enabling conditions, such as establishing new institutions or developing policy frameworks (Figure 5). As might be expected, this emphasis was especially prominent in EP 1, when key institutions (ONE, ANAE, ANGAP) to implement the NEAP were built (per the INTRODUCTION section). This focus reemerged in the post‐NEAP period when funding increased for institutional development and on‐site and species management and restoration (Figure 5). Although agreeing that funding for institutions and policies is crucial for conservation success, some of our informants worried that too much funding has gone to action plans with nothing to show afterward. “Most of the time the funding remains on paper (framework, governance, plans). There is often funding to develop action plans but not funding for the implementation of the project” (Civil servant, 2020).

Another interviewee went so far as to say that all projects start with a prestudy to make a strategic plan, instead of adapting existing ones and using funds for implementation. The result is typical of projectification: projects work in silos, starting from scratch, and miss opportunities to build on previous work and coordinate among relevant actors.

The greatest percentage of expenditure on PAs occurred during EP 2 (1997–2002) (Figure 5), showing an already strong focus on area‐based conservations before Ravalomanana's promise to triple the country's PA network in EP 3. Ravalomanana's ambitious conservation plans clearly spurred the interest of donors (Figure 2), who also increased support for law enforcement and prosecution activities (Figure 5). This finding accords with the aggressive antifire government agenda during EP 3, when strict bans on burning were announced and many swidden farmers were jailed (Kull, 2014).

Corson (2016) criticized the narrow biodiversity focus of conservation and development funding for Madagascar, detailing how a strong international conservation NGO lobbying in Washington led to this narrowing. Many Malagasy practitioners and civil servants we interviewed supported this critique, highlighting the lack of integrating conservation into a wider rural development agenda as a key reason for limited conservation success.

We put in place conservation to protect the forest but we do not put in place accompanying measures to replace the income of those who depend on the forest. Conservation limits access to resources and therefore leave no alternative for the local community. Recently the Ministry made an evaluation to determine the effectiveness of conservation in Madagascar. Was the creation of protected areas a solution to protect the forest? And the answer is NO, because conservation has not prevented deforestation, the rate of deforestation has even increased. Solutions are that we must always consider the humanitarian side, the social and economic development of the local community through their empowerment (Civil servant, 2019).

Our quantitative results suggest that this trend has continued in the post‐NEAP period (e.g., livelihood support did not increase) (Figure 5). However, interviews indicated that practitioners perceived funder interests had changed toward a wider recognition of the importance of rural development in conservation. An interviewee with long experience in conservation described changing funding fads in the following way: “First everyone did a lot of research, then Madagascar decided to expand the protected areas and suddenly everyone wanted to fund the establishment of PA's for 5‐6‐10 years, and now everyone wants to fund sustainable development i.e. small development projects on sites” (Malagasy NGO staff, 2019). This informant continued: “the funding follows the politics. For example, now the ministry is called the Ministry of Environment and Sustainable Development so more funding goes to development activities. The minister now wants reforestation, but in my opinion, the goal is not to improve conservation but to have more wood for exploitation.”

If there has been a shift toward more strongly funding rural development concerns in conservation (possibly in more recent years not captured in our quantitative analysis), it could have an effect on power relations among conservation‐relevant actors. We found some indication of this; some organizations with a strong PA mandate reported finding it more difficult to find funding. “Donor funding is directed more to address issues related to wars, diseases and poverty. French cooperation has not funded us for the last 5 years. Even though they invest in Madagascar they give their funding directly to activities that fight against poverty, not to environment. German cooperation is the only one who continues to fund us” (Malagasy conservation actor, 2019).

Our quantitative analyses showed a shift toward restoration projects at the end of our study period (seen as an increase in the land or water management category) (Figure 5), a point many interviews also made in describing a post‐NEAP focus on climate change and restoration. This trend is also reflected in president Rajoelina's forest restoration focus and tree planting initiatives, such as the African Forest Landscape Restoration Initiative (AFR100) and different tree planting campaigns. Some PA managers we interviewed criticized this shift as diverting the focus from stopping deforestation inside PAs. Many also saw risks associated with neglecting endemic species in favor of non‐native pine or eucalyptus. It remains unclear the extent to which non‐native tree planting might take place in PAs, but many conservationists expressed feeling pressure to start restoration activities due to the available funding. One manager commented:

The new government is all about planting trees. It is not bad, but they need to understand what is behind deforestation before saying that planting trees is a solution. There is no way that it could be the solution. It does not stop illegal logging or charcoal. It is just a little piece of a puzzle in stopping deforestation. But now they put pressure on protected area managers to plant trees whereas we want to protect already existing forests. We do not want to plant trees and introduce weird species (Malagasy NGO, 2019).

Relatedly, Madagascar was selected as a World Bank pilot country for REDD+ implementation, and this funding was allocated to some PAs for avoiding deforestation and also for reforestation and afforestation activities. The government decided that all REDD+ funding must go through the state, and Ministère de l'Environnement et du Développement Durable (MEDD) sees it as a potential way to find more sustainable funding for nature conservation. This decision has not been popular among all conservation NGOs. However, in combination with a wider landscape approach and tree planting initiatives outside PAs, it could be seen as a reassertion of government authority in the national conservation agenda. As one informant put it: “At least this ownership of the country, this leadership, a courage to say ‘no, we will do like this’—maybe that [is what is needed]” (Civil servant, 2019).

### Power of the NGO sector and unclear role of the state

The conservation sector in Madagascar operates through a heterogeneous group of organizations—nonprofits and for profits, foreign and Malagasy, parastatal and private, big and small. When trying to tease out from official records the recipients of biodiversity funding, we found that the recipient or implementer was unknown or poorly reported in many cases. This dimension had more unknown codes than any other topic in our database (34% unknown for recipient sector and 65% unknown for recipient type). Keeping this uncertainty in mind, we found that the main recipients of biodiversity aid were the government of Madagascar and non‐Madagascar‐based NGOs, with funding distributed roughly equally between Madagascar‐based and non‐Madagascar‐based recipients (Appendix S8). The share of funding to Madagascar‐based recipients remained constant across different phases of the NEAP, but the government's share varied significantly from 1.33% in the first phase, to 74% before a political coup, and to 48% in the post‐NEAP phase (Appendix S8). Over time, the diversity of recipients increased, especially in the post‐NEAP period, with intergovernmental organizations and the education sector receiving increased funding (Appendix S8).

When discussing aid recipients with interviewees, Malagasy conservation practitioners, in particular, claimed that donors prefer INGOs over local NGOs, even when the latter has greater presence at conservation sites. “We have noticed that the donors don't have trust on national NGOs. When there is a competition, they choose the international ones” (Malagasy NGO staff, 2019).

Comparing themselves with big INGOs, staff from Malagasy NGOs and associations felt frustrated because they did not have international headquarters that could help smooth gaps in funding or enough staff dedicated to write funding applications. They also reported feeling pressure to partner with INGOs. “The small Malagasy associations have to start through small contracts with the big INGOs. And this is a problem too” (Malagasy NGO staff, 2019). Another informant explained: “But they [Malagasy NGOs] don't really exist. Those that are there, they are manipulated by the big INGOs. They give a bit of financing to manage a small protected area so they are satisfied” (Civil servant, 2019).

Many interviewees agreed that the government of Madagascar has very little control over the use of biodiversity funding. Donors are reportedly reluctant to fund state institutions directly, arguing that the risk of corruption is too great. According to our informants, this was the reason for the creation of parastatal ANGAP and MNP (by the World Bank and USAID) that now functions based on foreign funding and tourism revenues. One interviewee also suggested that the creation of the country's largest trust fund was based on a similar argument. “FAPBM was created with the influence of big international NGOs. They said that the state is corrupt and lacks management capacity. That is an excuse. They wanted an independent actor; they excluded the state” (Civil servant, 2019).

Some of the informants were critical of the decision to open PA governance rights to NGOs, surmising that it led to much of the peak funding after President Ravalomanana's Durban speech to be channeled primarily to INGOs. “SAPM [the Madagascar Protected Area System] was put in place to let also other NGOs to directly manage protected areas. All of this is done so that the foreigners could manage everything in Madagascar, so that Malagasy organization could not manage protected areas alone, and it was also a way of creating business” (Civil servant, 2019). An MEDD representative also believed that INGOs continue to be the main recipients of biodiversity funding to Madagascar. Some interviewees noted that this arrangement has marginalized the state: “The government feels of course a bit left aside…” (INGO staff, 2019).

Our interviews showed two strategies the government has used to assert or reassert their influence in conservation. First, in 2018, the government banned selling carbon credits from Madagascar by any other entity than the state (see also Streck [2020]). Since then, REDD+ work has been managed by the National Office for Climate Change and REDD+ (under MEDD). This agency is responsible for the contracting and validation of carbon credits, consistent with Malagasy Forest Policy, and for the distribution of funds to stakeholders. This was taken as a nationalist move by the international conservation actors, some of whom lost a direct funding source and attempted to lobby the government to reverse the decision.

Second, since 2020 the state has embarked on an approach referred to as *green diplomacy*, according to a ministry representative. MEDD now requires every conservation NGO to report to the ministry. This “means that all NGOs who work in Madagascar align themselves with the policy of the state about conservation and sustainable development” (Civil servant, 2022). However, with minimal public funding, the ministry and ONE have depended on project funding and collaborations with international donors. This arrangement underscores how fixed‐term project funding challenges state capacity to carry out legal basic functions and develop a workable long‐term strategy.

Generally, interviews revealed a picture of weak environmental governance in Madagascar, marked by a lack of funding for basic administrative functions and law enforcement despite decades of international funding. One practical consequence is that PA management over time has been difficult. “The conservation NGOs know what they want to do, and they try to do it. But it is not only up to conservation NGOs; governmental decision and application of laws is also needed and that needs funding at a bigger scale” (INGO staff, 2019). The interviewees in government offices expressed frustration because they perceived that most funding was being consumed by foreign partners to pay for international consultants or to develop rather than implement policy frameworks. A consensus emerged that funding has not helped solve structural problems and has led to a feeling of dependency. “If you try to do politics you have to have power over the funds as well,” as a civil servant put it (2019). Another Malagasy interviewee concluded, “Why is there no development in Madagascar after all these decades of independence? Because Madagascar has never been truly independent. This influences our policy even in terms of biodiversity. Biodiversity is not our priority! We have good environmental legislation but we cannot enforce it because of the politics” (Malagasy conservation actor, 2019).

## DISCUSSION

Despite Madagascar's role as a biodiversity hotspot and as a major recipient of international biodiversity funding (Miller et al., 2013), we found that international aid earmarked for biodiversity conservation (nearly US$1 billion from 1990 to 2018) was only at average levels compared with other aid‐receiving countries. Biodiversity aid represented 4.25% of all aid received, whereas in 2019, for example, such aid was estimated to be 2.6–6.3% of total ODA in aid‐receiving countries (our calculations based on Deutz et al. 2020 and OECD 2024b).

However, this average obscures variations and an overall decline in biodiversity aid over the study period. Political instability, such as the political coup in 2009, clearly affected donor willingness to operate in Madagascar. These results align with previous literature showing that good governance was associated with increased conservation aid (Miller, 2014; Miller et al., 2013; Reed et al., 2020). The most prominent change in relative funding provided by different donors was the diminishing role of the United States and the increasing role of Germany.

Our results charted changes in the kind of conservation actions being funded, and these patterns along with interviews suggest that they may reflect not only donor interest but also attempts by the Malagasy state to exert more influence over conservation in the country. Renewed emphasis on support for institutional development in the post‐NEAP period (2009–2018) seemed linked to the recent focus on reforestation and tree planting, possibly in an attempt by the state to capitalize on REDD+ financing and regain some control in the sector, which has so far been heavily driven by donors and international NGOs (Corson, 2016; Méral, 2012). Some argue that carbon market financing moves decision‐making outside the country and is yet another instrument to sideline the state (Méral et al., 2009; Moïse, 2014). Considering this, our results suggesting that in recent years there has been an active attempt by the state to take control of the funds are particularly interesting.

In line with larger trends since the 1990s, we found evidence of short termism and an increasing projectification in the conservation sector in Madagascar. Funding flows followed short cycles, and conservation practitioners expressed concerns of needing to rebrand core operations and actions as short‐term projects to compete for international funding and grants. One consequence of this faddism, which is characteristic of the conservation sector more generally (Redford et al., 2013) and the wider development sector (Hicks et al., 2008), is missed opportunities for learning, which may help account for suboptimal conservation results. Another is lack of funding for basic ongoing management costs (Méral et al., 2009). In the case of PAs, our results suggest that funding fluctuation and uncertainty weakened trust and long‐term relationships with local communities. In contrast, the overall shift away from funding PAs could be seen as an attempt to integrate conservation within wider environmental and rural development concerns. Yet, the impact of this change remains unclear, with recent funding criticized as still focused on short‐term development projects and on overreliance on market‐based approaches that leave power in the hands of foreign actors (Méral et al., 2009; Moïse, 2014).

How these shifts have affected the role of the state remains somewhat unclear. Informants confirmed the view that many funders might have wanted to bypass the state because it has been perceived as corrupt and inefficient (Méral et al., 2024). Although USAID worked in 1990s to establish institutions, such as MNP and ONE, the diminishing role of the United States in conservation has meant less funding for ONE, with MNP now competing for funds with other conservation NGOs. Corson (2010) showed that the parallel trends of neoliberalization and a sole focus on biodiversity aspects within the environmental agenda have put power in the hands of nondemocratically elected, foreign actors (such as INGOs). These trends toward weakened state institutions can jeopardize conservation success because state actors no longer will be able to run state institutions and enforce laws effectively, a concern shared by many of the informants we talked to. Yet, our quantitative analyses identified the government as one of the main recipients of conservation funding in Madagascar. The idea of a weak state that passively watches as the funds are being redirected is not supported by our results (as found elsewhere in Africa [Ongolo et al., 2015]). On the contrary, the qualitative and quantitative results of our analyses showed that the post‐NEAP governments sought to maintain control of REDD+ funding and stipulated that carbon market activities must go through the state, which can be seen as a way to take back control from foreign actors.

### Policy implications

The Kunming–Montreal Global Biodiversity Framework (GBF) provides important context for our findings and the future of biodiversity funding in Madagascar and beyond. Funding will have an important influence on the realizing of GBF targets, such as protecting 30% of terrestrial and marine areas and increasing restoration efforts by 30% by 2030 (Convention on Biological Diversity, 2022). Countries have agreed to mobilize $200 billion annually to support global conservation, but this commitment only includes $20–30 billion for low‐ and middle‐income countries (Gilbert, 2022), which have typically been the most underfunded in terms of conservation (Waldron et al., 2013). Achieving the area‐based 30×30 target has been estimated to cost US$178 billion annually (Waldron et al., 2020) and thus risks consuming the lion's share of committed funds. However, these commitments remain well below the US$967 billion estimated to be needed annually to reach all of GBF targets (Karolyi & Tobin‐de la Puente, 2023).

The extent to which the new commitments and modalities (e.g. the GBF Fund) will address issues raised in our analyses for Madagascar and other low‐ and middle‐income countries remains unclear. Will short‐term projects still be the main operational mode or can aid commitments support long‐term development? Grantee interviewees argued that more streamlined funding mechanism could decrease the time needed in applying for and reporting on smaller projects and thus ease the administrative burden. However, will smaller actors, such as local NGOs, Indigenous peoples, and local community organizations, have any chance of securing such funding or will they be increasingly dependent on INGOs or on operating as their subcontractors? To what extent will there be funding for ongoing management activities, which are crucial for long‐term outcomes, as stated by our informants and independent studies (Eklund, Jones, et al., 2022; Gill et al., 2017)? Recognizing structural challenges in a given country's conservation sector and the different interests involved will be crucial to determining funding needs and potential leverage points for advancing long‐term biodiversity goals. To aid future analyses and advance these objectives, more extensive and consistent reporting across donors on geographic and recipient information is required.

### Limitations and paths forward

International aid agencies have attempted to standardize reporting requirements and even track specific sectors more reliably through sector codes and different markers. However, we learned that the biodiversity markers were not always used in the reporting, thus making it difficult to detect the projects that had a likely biodiversity conservation component included. The systematic protocol we developed to detect biodiversity aid should mitigate this risk, but it is still possible we missed or misclassified some funding flows. Insufficient information reported by donors prevented us from identifying especially funding recipients and getting further insights to analyze donor–recipient linkages. An improved and standardized reporting would increase transparency of the impacts of conservation funding and enable in‐depth analysis, a recommendation that has been raised for conservation expenditures more generally (Iacona et al., 2018). We tried to geolocate as much of the funding flows as possible, but doing so provided little insight because the geographical resolution was typically too coarse to allow for any interesting further analyses.

China has emerged as an important player in international aid (Brazys & Vadlamannati, 2021). However, China did not report its funding in the OECD data we used as the basis for our analyses. Newly released data (Custer et al., 2023) indicate that the amount of Chinese aid to Madagascar (for all development activities, possibly including biodiversity‐related ones) was equal to 6% of the overall reported aid to the country in 1990–2018, possibly placing China on par with other donors, such as Japan, Korea, and the African Development Fund, in terms of financial influence in Madagascar. Future studies should therefore include an inspection of these new data to better infer the role China might have had in the biodiversity sector. Notably, China was never mentioned in our interviews.

As this study focused on ODA, funding from the private sector and philanthropies was not included. We estimate that only a small share (4%) of biodiversity funding to Madagascar originates from philanthropic foundations, as per funding reported in the Foundations Center database (Candid, 2025). The role of private foundations and other philanthropic organizations compared with bilateral and multilateral donors merits further investigation (Gruby et al., 2023). The same applies to private sector funding for biodiversity, which can be substantial yet difficult to trace except for special cases (Devenish et al., 2022).

Despite these limitations, our study remains relevant and highlights important findings beyond Madagascar. If the world is serious about saving biodiversity, funding needs to be ramped up drastically and be much better targeted. For the majority of countries with high biodiversity situated in the Global South, this will mean a heavy reliance on aid, and thus efforts are needed to develop the funding structures in accordance with local needs (Eklund, Cheek, et al., 2022). We believe our mixed‐methods approach and systematically developed protocol for identifying and categorizing aid data have much to offer for future research to this end in Madagascar and beyond.

## Supporting information



Supporting information
