# Supplementary material for: Influence of funding fads and donor interests on international aid for conservation in Madagascar
Source: Conserv Biol. 2025 Aug 15;39(6):e70122. doi: 10.1111/cobi.70122 (PMC12658932; doi:10.1111/cobi.70122)
Supplement: Supplementary file 1 — Supporting information [file COBI-39-e70122-s001.docx]

Supporting Information

Appendix S1. Calculations with deflators

The OECD Data was downloaded as USD Constant 2018, so only the current (nominal) amounts in AidData, Foundation Center data and Macarthur data needed to be converted to USD constant 2018, which was done using deflators. We used the ready OECD DAC deflators, but for nine missing countries (Estonia, India, Kuwait, Liechtenstein, Monaco, Saudi Arabia, South Africa, Thailand, and United Arab Emirates) we used the method by **Steward & Russel 2015.**

Appendix S2. Details on the coding protocol for inclusion in funding database

We originally categorized the cases (i.e. a funding commitment appearing in the funding database) into four categories: 1) Biodiversity related funding, 2) Non-biodiversity related funding, 3) unclear biodiversity implications, and 4) Insufficient information. Category 3 denotes cases where the implications (direct or indirect) for biodiversity conservation were unclear based on available information whereas category 4 included cases where there was simply not enough data to make any inferences. However, in presenting the results, these two categories were combined as “Unclear/Insufficient information”.

We reviewed data from each CRS-sector independently to make final determinations on inclusion in our funding database according to the following coding protocol:

1. If Rio marker reported biodiversity as a principal/significant objective, the listing was included as biodiversity related.
2. If Rio marker mentioned climate, environment, or desertification as an objective, the listing was either screened manually (for the following sectors: education, health & population, water, government, energy, agriculture, tourism, environment, multisector, general budget support, reconstruction relief and rehabilitation, disaster prevention  & preparedness, unallocated), or with the search word list (for the following sectors: health & population, other social sectors, transport & storage, communications, banking & business, mining & construction, development food assistance, action related to debt, emergency response, administrative costs of donors, refugees in donor countries) and if it gave a hit, then it was manually checked. If no hits, the listing was coded as non-biodiversity related.
3. If the listing contained no Rio marker, the cases were still checked with the search word list (Appendix S1), and any listing in the following sectors were manually inspected in order to minimize the risk of missing biodiversity related funding: agriculture, tourism, environment, multisector, general budget support, unallocated.

All cases coded as 1 (biodiversity related) were manually checked, including those that were “automatically” coded as 1 due to their biodiversity marker. To minimize the risk of “losing” biodiversity related funding (I.e. coding it as non-biodiversity related), all the cases coded as 2 or 3 or 4 were checked again with the search words (Appendix S1). Finally, all cases coded as 2, 3 and 4, were double checked by OECD and AidData purpose codes. The cases listing biodiversity, environmental education/training, environmental policy, administration and management, environmental research and general environmental protection were checked manually once more to detect if some biodiversity-related cases would have gone unnoticed.

**Appendix S3.** List of search words in different languages for screening the funding database.

| **English** | **French** | **German** | **Italian** | **Spanish** | **Dutch** |
| --- | --- | --- | --- | --- | --- |
| Biodiversity | Biodiversité | Biodiversität | Biodiversità | | biodiversidad |
| Bio-diversity | Bio-diversité | Bio-diversität | Bio-diversità | bio-diversidad | Bio-diversiteit |
| Diversity | diversité | Vielfalt | diversità | diversidad | diversiteit |
| Fauna | Faune |  |  |  |  |
| Flora | Flore |  |  |  |  |
| Forests | forêts | Wälder | Foreste | Bosques | Bossen |
| Rainforest | Forêt tropicale | Regenwald | Foresta pluviale | Selva | Regenwoud |
| Forest | Forêt | Wald | foresta | Bosque | Woud |
| Forestry | sylviculture | Forstwirtschaft | silvicoltura | silvicultura | bosbouw |
| reforestation | reboisement | Wiederaufforstung | rimboschimento | repoblación forestal | herbebossing |
| Ecoregion | Écorégion | Ökoregion | Ecoregione | Ecorregión | Ecoregio |
| Nature | La nature | Natur | Natura | Naturaleza | Natuur |
| Natural | naturel | natürlich | naturale |  | natuurlijk |
| national park | Parc national | Nationalpark | Parco Nazionale | Parque Nacional | Nationaal Park |
| Protected area | Zone protégée | Geschützter Bereich | Area protetta | Área protegida | Beschermd gebied |
| Reserve | réserve | Reservieren | Riserva | reserva | Reserveren |
| Park | Parcs |  | Parchi | Parques | Parken |
| Conservation | Préservation | Erhaltung | Conservazione | Conservación | Behoud |
| Protection |  | Schutz | Protezione | Proteccion | Bescherming |
| Preserve | Préserver | Erhalten | Conserva | Preservar | Behouden |
| Species | Espèce | Spezies | Specie | Especies | Soorten |
| Environmental | environnemental | Umwelt | ambientale | ambiental | milieu |
| Biological | Biologique | Biologisch | Biologica | Biológico |  |
| Ecosystem | Écosystème | Ökosystem | Ecosistema | | Ecosysteem |
| Ecosystem-based | Basé sur l'écosystème | Ökosystembasiert | Basato sull'ecosistema | Basado en ecosistemas | Ecosysteem-gebaseerd |
| Ecotourism | Écotourisme | Ökotourismus | Ecoturismo | | Ecotoerisme |
| Eco-tourism | Éco-tourisme | Öko-tourismus | Eco-turismo | |  |
| Biosphere | Biosphère | Biosphäre | Biosfera |  | biosfeer |
| Wildlife |  | Tierwelt | Fauna selvatica | Fauna silvestre | Dieren in het wild |
| Ecology | Écologie | Ökologie | Ecologia | Ecología | Ecologie |
| Ecological | Écologique | Ökologisch | Ecologico | Ecológica | Ecologisch |
| Hotspot | Point chaud | | Punto di accesso | |  |
| Coral reef | récif de corail | Korallenriff | barriera corallina | Arrecife de coral | koraalrif |
| Bat | Chauve souris | Schläger |  | Murciélago | Knuppel |
| Chameleon | Caméléon | Chamäleon | Camaleonte | Camaleón | Kameleon |
| Gecko |  |  | Geco |  | Gekko |
| Fossa | Fosse |  |  | Fosa |  |
| Lemur | Maki |  | Lemure | Lémur |  |
| Turtle | Tortue | Schildkröte | Tartaruga | Tortuga | Schildpad |
| Whale | Baleine | Wal | Balena | Ballena | Walvis |
| Mangrove |  |  | Mangrovie | Mangle |  |
| Fisheries | Pêche | Fischerei |  | Pesca | Visserij |
| Drylands | Terres arides | Trockengebiete | | Tierras Secas | Droge gebieden |
| Wildlands | Terres sauvages | Wildnis |  | Tierras salvajes | |
| Wilderness | Région sauvage | | Natura selvaggia | Desierto | Wildernis |
| Ecoagriculture | Écoagriculture | Ökoagriculture | Ecoagricoltura | Ecoagricultura | |
| Ecodevelopment | Écodéveloppement | Ecoentwicklung | | Ecodesarrollo | Ecologische ontwikkeling |
| Landscape | Paysage | Landschaft | Paesaggio | Paisaje | Landschap |
| Land tenure |  |  |  | Tenencia de la tierra | Grondbezit |
| Land management | La gestion des terres | Land verwaltung | Gestione del territorio | Gestion de tierras | Landbeheer |
| Resilience | Résistance | Elastizität | Resilienza | Resiliencia | Veerkracht |
| Seascape | Paysage marin | Seelandschaft | Paesaggio marino | Marina | Zeegezicht |
| Afforestation | Boisement | Aufforstung | Imboschimento | Repoblación forestal | Bebossing |
| CBC |  |  |  |  |  |
| CBNRM |  |  |  |  |  |
| CBWM |  |  |  |  |  |
| ICDP |  |  |  |  |  |
| ICZM |  |  |  |  |  |

**Appendix S4.** Conservation actions according to the IUCN classification system v. 2016. We used the main categories for coding the funding projects as details were often lacking to be able to infer subcategories.

| Code | Name | Description |
| --- | --- | --- |
| 0 | Unclear, unspecified | Not enough information to determine conservation actions |
| 1 | Land/water management | Actions directly managing or restoring sites, ecosystems and the wider environment. |
| 11 | Site/area stewardship | **Enhancing viability / mitigating stresses** for sites and/or ecosystem targets, especially on a smaller scale |
| 12 | Ecosystem & Natural process (re)creation | **Restoring** missing or severely degraded ecosystems and ecosystem functions and processes, especially on a large scale |
| 2 | Species management | Actions directly **managing or restoring specific species** or taxonomic groups. |
| 21 | Species stewardship | **Enhancing viability of / mitigating stresses to specific taxa** within their current range. |
| 22 | Species Re-introduction & translocation | **Transferring species or genetic material** to places where they formerly occurred or to suitable future habitat or benign introductions of species to an ecosystem |
| 23 | Ex-situ conservation | **Protecting specific taxa** in artificial settings with the aim of ultimately restoring them to their natural settings. MÄ: add here genetic preservation (e.g. seed banks) |
| 3 | Awareness raising | Actions making people aware of key issues and/or feeling desired emotions, leading to behavior change. |
| 31 | Outreach and communications | Promoting desired awareness and/or emotions and subsequent behavior change by providing information to target audiences through appropriate channels. Includes "training" or "education" that is primarily designed to get folks to change a behavior (educating homeowners to plant native rather than non-native species), but trainings that teach specific skills (how to do controlled burns) fit in 9.2 Training & Capacity Development. Confrontational outreach actions go in 3.2 Protests & Civil Disobedience. **Raising awareness** via reported **media** (newspapers, TV, radio, curated blogs), social media (Facebook, personal blogs), ads & marketing (mail campaign, celebrity media ads), displays (museum or zoo exhibits, park signs), **art** (paintings, recorded music), **performances** (puppet shows, theater), **person-to-person engagemen**t (info booth, peer mentor), experiential learning (nature walks, outdoor education). |
| 32 | Protest and disobedience | Promoting desired awareness and subsequent desired behavior change by conducting protests, naming and shaming, civil disobedience, or sabotage activities. Activities that seek to draw attention to and/or impede various conservation threats or drivers of threats. In general, these activities seek to put pressure on the actors responsible for the threats and/or make it too expensive to continue with the threat behaviors. Note that inclusion of illegal actions in this classification explicitly does NOT constitute an endorsement of these tactics. |
| 4 | Law enforcement and prosecution | Actions monitoring and enforcing compliance with existing laws and policies at all levels to deter threats or compel conservation action. |
| 41 | Detection and arrest | **Detecting, directly stopping, and/or deterring violations of existing laws and policies**. Interdiction activities are designed to both **stop existing law breakers** as well as **deter future law breakers** from illegal and generally criminal activities. Interdiction activities can take place at any point along the transactional chain that links resource harvesters or poachers, traders, financers or other middlemen, and end consumers. Reducing or deterring illegal behaviors through surveillance, patrolling, guarding checkpoints/borders, carrying out investigations, establishing/maintaining informer networks, arrest & interdiction. |
| 42 | Criminal prosecution and conviction | Ensuring appropriate application of sanctions for violations of existing laws and policies. Activities designed to ensure that **appropriate sanctions are meted out** and that laws will thus have the desired deterrence effect. However, actions that are primarily designed to teach prosecutors how to do their jobs could go in 9.2 Training & Capacity Development and actions that are primarily designed to create or improve courts or prisons could go in 10.2 Institutional & Civil Society Development since these are more enabling condition actions. Deterring threat behaviors through prosecuting alleged crimes, trying alleged crimes, punishing proven crimes (prisons, fine collection, rehabilitation). |
| 43 | Non-criminal legal action | Threatening or bringing non-criminal legal action to get individuals, organizations, agencies or firms to change or deter undesired behaviors or compel conservation action. Non-criminal legal activities designed to change or deter undesired behaviors or compel desired conservation action. Activities can be initiated by relevant government agencies using their statutory authority, or by various actors through civil legal proceedings. Includes processes in which **government environmental agencies are authorized to officially review or comment on policies or projects** (e.g. highway construction, new housing developments). Changing behavior through **civil law suits, agency enforcement, agency or judicial review implemented by other agencies or private sector actors**. |
| 5 | Livelihood and economic and moral incentives | Actions using livelihood, other economic and moral incentives to directly influence attitudes and behaviors |
| 51 | Linked enterprises & alternative livelihoods | **Developing enterprises that directly depend on the maintenance of natural resources or provide substitute livelihoods** as a means of **changing attitudes and behaviors**. Both linked enterprises and alternative livelihoods involve providing income and/or subsistence resources to natural resource users. Linked enterprises depend on the natural resource base and thus provide an incentive to local stakeholders to use these resources sustainably over the long-term. **Alternative livelihoods attempt to find a substitute for a damaging resource-based livelihood** (e.g. unsustainable fishing or logging). Creating incentives to change behaviors through linked product-producing enterprises (consumptive), linked ecological service-using enterprises (non-consumptive), non-linked enterprises & livelihoods. Examples: **non-timber forest product** harvesting business, **wild salmon fishery**, **subsistence hunting & gathering**, **training loggers to be ecotourism guides**, **training loggers for factory jobs.** |
| 52 | Better products & management practices | Developing, promoting and/or **providing more environmentally-friendly products or practices** that substitute for environmentally damaging ones. This category includes finding substitutes for environmentally damaging products and behaviors, and to encompass environmentally better products and management practices. Although many people refer to "best" management practices, we use the term "better" to show that practices can always be further improved. There is some overlap with 5.3 Market-Based Incentives since certification systems are often designed to incent or promote better management practices among service providers or commodity producers. This category, however, is more about developing, promoting, providing, and/or removing barriers (e.g. risk minimization) to adoption of better products and practices. |
| 53 | Market-based incentives | Using market mechanisms to change behaviors and attitudes. Creating incentives to change behaviors through "Green" **certification of products or services**, **boycotts** of "non-green" products or services, **environmental markets** (e.g. **CO2 emissions**, water), "**Green" financing** (e.g. bank loans, divestiture). Examples: promoting responsible palm oil production, educating consumers about seafood sustainability, boycotts of non-dolphin safe tuna, creating carbon market for forest conservation, providing loan guarantees to green businesses, campaign to get universities to divest from fossil fuel companies. |
| 54 | Direct economic incentives | **Using direct or indirect payments** or ascribing economic value to change behaviors and attitudes. Activities using non-market based financial and economic incentives to change behavior. In this case, the implementer has to raise ongoing funds needed to make conservation payments or provide subsidies and also needs to ensure that the subsidies go to the right people. It is also possible to have financial **dis-incentives in the form of taxes**. There may be a research component to Valuation of Ecological Services. **Awards & prizes** could also be seen as an outreach strategy, since they often seek to draw attention to conservation work and issues as much or more than they directly incent conservation behaviors. |
| 55 | Non-monetary values | Using intangible and moral values to change behaviors and attitudes. Creating incentives to change behaviors by appealing to **health & social service benefits, security benefits, spiritual / moral / cultural benefits.** |
| 6 | Conservation designation and planning | Actions directly protecting sites and/or species. |
| 61 | Protected area designation &/or acquisition | Legally or formally **establishing or expanding public or private parks**, **reserves, and other protected areas** roughly equivalent to IUCN Categories I-IV. |
| 62 | Easements and resource rights | Legally or formally e**stablishing protection of some specific aspect** of the natural resources on public or private lands. |
| 63 | Land/water use zoning and designation | Legally or formally establishing protection of some specific aspect of the natural resources on public or private lands |
| 64 | Conservation planning | **Planning for management of sites, species, or thematic conservation projects.** |
| 65 | Site infrastructure | Building or maintaining the **physical infrastructure** for protected areas and other conservation sites. Building or maintaining infrastructure to support site through protection (e.g. border fences, patrol huts), direct management (e.g. greenhouses, supply sheds), resource extraction (e.g. sawmill, fish processing building), transport (e.g. roads, airstrips), tourism & recreation (e.g. visitor centers, boat ramps), learning & research (e.g. student dorms, labs), administration (e.g. headquarters, power plants). |
| 7 | Law and policy frameworks | Actions **developing and influencing legislation, policies and voluntary standards** affecting conservation. |
| 71 | Laws, regulations & codes | Creating, amending, or influencing **laws, regulations and codes at all levels** |
| 72 | Policies & guidelines | Creating, amending, or influencing **policies and guidelines** at all levels MÄ: frameworks |
| 8 | Research and monitoring | Actions collecting data and transforming it into information to support conservation work. |
| 81 | Basic research & status monitoring | **Collecting, managing and analyzing data and creating information** about any conservation-related factors |
| 82 | Evaluation, effectiveness measures & learning | Assessing and learning about the **effectiveness of conservation work**. |
| 9 | Education & training | Actions enhancing the knowledge and skills of specific individuals. |
| 91 | Formal education | Enhancing knowledge and skills of students in a **formal degree** program. Formal education seeks to build the long-term conservation capacity of students. Providing conservation courses, modules or materials for primary, secondary, college or university education, as well as adult/continuing education. A specific strategy of providing general education to resource users so that they have non-resource using livelihood alternatives should be included in domain 5 under linked enterprises & alternative livelihoods. Examples: creating a high school environmental course, teaching a conservation module in a university course, writing a conservation textbook. |
| 92 | Training & individual capacity development | Enhancing **knowledge, skills and information exchange** for **practitioners, stakeholders, and other relevant individuals** in structured settings **outside of degree programs**. Coaching involves more hands-on work with practitioners as they actually implement conservation activities (which falls within domain 5.2 Better products and management practices) whereas training is more about providing basic knowledge and skills that will be applied at a later date. There is a fine line between providing technical assistance as primary strategy vs providing technical assistance as one activity within another action. Providing conservation capacity development through **hands-on coaching & technical assistance**, **workshops & professional development training courses, developing training materials** (manuals, software, videos). Examples: coaching a team developing a strategic plan, providing technical assistance to landowners to use better practices, training course in proscribed fire, writing how-to manuals for project managers, **elders sharing traditional ecological knowledge.** |
| 10 | Institutional development | Actions creating the institutions needed to support conservation work. |
| 101 | Internal organizational management & administration | The basic work needed to **establish and operate** conservation organizations. Actions like **hiring and managing staff** for protected areas or conservation agencies, serving on the board of a conservation organization, **managing a conservation program**, providing basic support functions for a reserve. Establishing & managing conservation organizations through governance, executive management, human resources, financial & legal management, **fundraising, communications, program / project management, provision of org facilities & technology, support functions** (secretaries, guards, drivers). |
| 102 | External organizational development & support | Creating or providing **non-financial support & capacity building** for conservation organizations. There is a fine line between providing technical assistance (9.2 Training & Capacity Development) which focuses primarily on individuals and project teams versus this category which focuses primarily on entire programs and organizations. Establishing & supporting organizations through direct organizational support (**consulting / volunteering**), organizational establishment & incubation, providing association / membership services, developing / providing organizational **management tools**. Examples: providing consulting services to a conservation organization, **international volunteers** or circuit riders helping to develop organizational capacity, work to create and strengthen courts that can prosecute wildlife crimes, helping catalyze and incubate formation of a new land trust, developing work planning software for conservation organizations. |
| 103 | Alliance & partnership development | **Forming and facilitating partnerships, alliances, and** networks of organizations. This category encompasses work to create cross-organizational conservation institutions. Many of these partnerships provide support to their members so there is a fine line between 10.2 External Organizational Development & Support and this category; the former has supporting individual organizations as its primary focus whereas the latter is more about maintaining the network. Examples: convening meetings of local stakeholders in a community reserve, an international forum to share information about wildlife crimes, a membership services association of land trusts, a regional learning network, an international academic society. |
| 104 | Financing conservation | **Raising and providing funds for conservation work**. This category includes work aimed primarily at providing funding for conservation work. Obviously, almost every other action has some component of financing. There is some potential overlap with 5.4 Direct Economic Incentives and this category; with the difference being that the former is targeted to incenting specific behaviors, whereas the latter is about generally providing the funds required to take on other conservation actions. Providing funds for conservation including member / small contributions, unrestricted grants, restricted grants (e.g. **scholarships, funds for a specific project**), **program related investments / soft loans, commercial** loans, in-kind services, financing mechanisms (e.g. debt-for-nature swaps). Examples: door-to-door fundraising efforts, private foundation or government grants, foundation investments in green businesses, corporate philanthropy, national **debt-for-nature swaps**. |

**
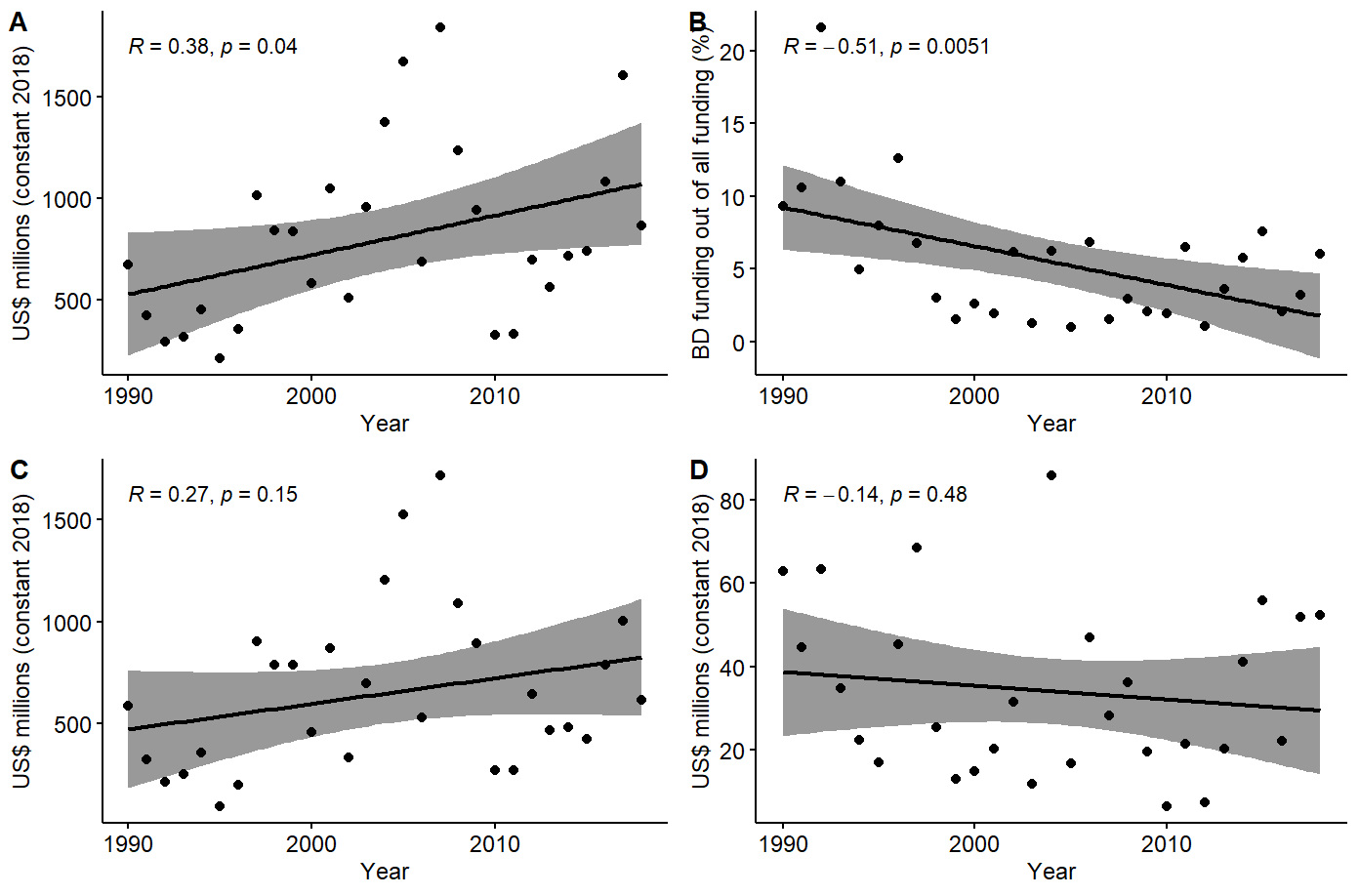
**

**Appendix S5.** Scatterplots with Pearson correlation coefficients and 95 % confidence intervals. A) amount of all development aid across the years 1990-2018, B) Percentage of biodiversity funding out of all funding across the years 1990-2018, C) amount of nonbiodiversity funding across the years 1990-2018, D) amount of biodiversity related funding across the years 1990-2018.

EP3

EP1

US millions (constant 2018)

Share of biodiversity funding of all funding (%)

**Appendix S6.** Biodiversity-related funding (blue line, in millions of constant 2018 USD) and share of total biodiversity funding (orange line) over time, after excluding US funding (dotted lines, linear time trend; rectangles, conservation policy periods described in Introduction; EP1, 1990 to 1996 environmental program 1; EP2, 1997-2002 environmental program 2; EP3, 2003-2008 environmental program 3; Post-NEAP, 2009-2018 post-National Environmental Action Plan).

**Appendix S7.** The top 10 donors and their share of the overall development aid to Madagascar between 1990 and 2018.

| **ALL FUNDING** |  |
| --- | --- |
| **SUMMARY (TOP 10)** | |
| **Donor** | **% of total of total** |
| **World Bank - International Development Association (IDA)** | 21.00835864 |
| **European Communities (EC) / European istitutions** | 15.53454496 |
| **France** | 12.52366639 |
| **United States** | 8.534166899 |
| **Japan** | 6.073444269 |
| **African Development Fund (AFDF)** | 5.406892545 |
| **Korea** | 5.269189284 |
| **Germany** | 4.241048425 |
| **International Monetary Fund (IMF)** | 2.64085718 |
| **Italy** | 2.001056998 |
| **Others (56 donors)** | 16.76677441 |


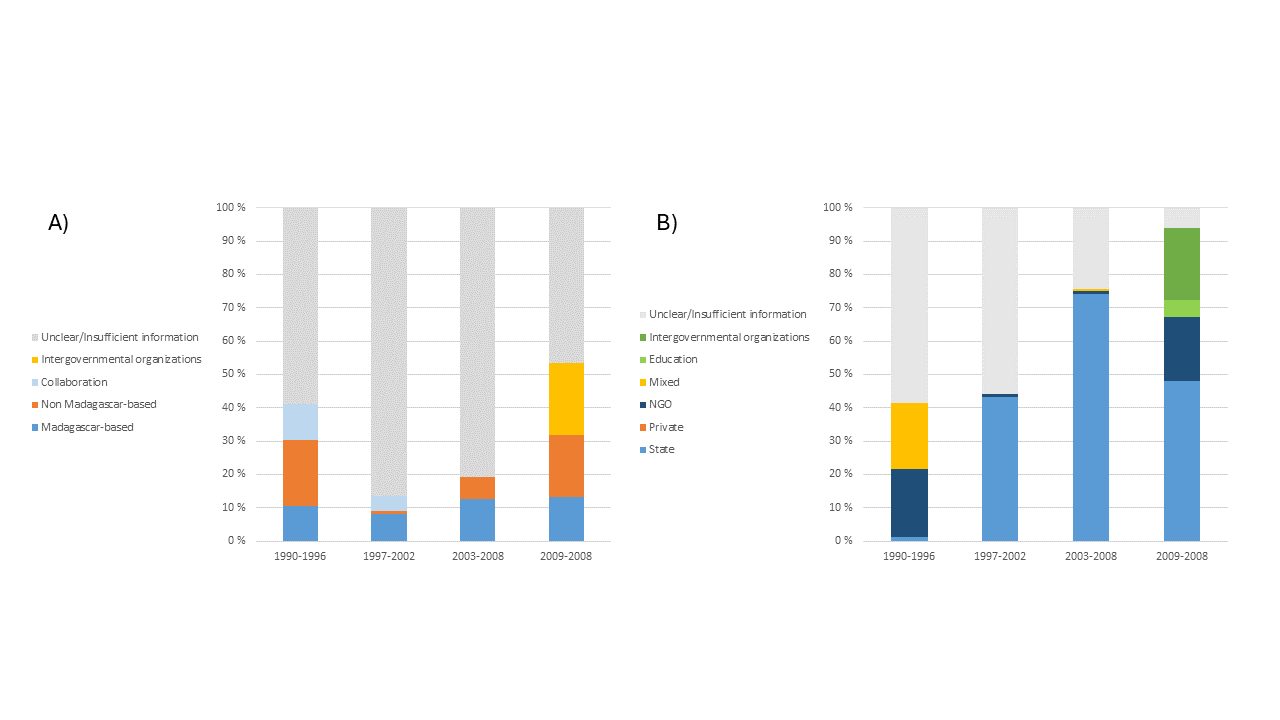


**Appendix S8.** Difference in A) recipient type, and B) sectors they operate in, over the four policy periods inspected. Percentages indicate the share in funding amounts.

**Appendix S9**. Interview protocol

Open-ended questions for interviewing conservation practitioners in Madagascar

**Conservation background in the target country:**

1. For how many years have you been involved in biodiversity conservation/environmental protection efforts at any scale in Madagascar?
2. What is your current role (or what are your duties)? Where are you based now? How big is the organization? How many employees in Madagascar?
3. For what other organizations within the conservation/environmental management sector have you worked?

**Experience with different types of funding**

1. During your time in the conservation/environmental sector, what funding instruments do you have experience with? *(e.g. government budget allocations, traditional short-term donor project funding, conservation trust funds, payment for ecosystem services, REDD, entrance fees)*
2. What funding sources/different funders do you have experience from/have you worked with?
3. Have you detected trends in what the funders like to fund and how has that changed over time?

*(only mention the following examples if they are not giving any answers at all: e.g. shift from fortress conservation to community based conservation, shift from terrestrial to marine etc.)*

1. In your organization, what is the proportion of funding from international donors versus national funding from the Malagasy government?
2. In your experience, what type of funding has worked best? Why and how?
3. In your experience, what type of funding has been most difficult or least successful? Why and how?
4. Do you/your organization work in association to some protected area? If so, which?

If trust funds does not come up in answers to the previous questions, ask:

- Do you have experiences with funding from Conservation Trust Funds in Madagascar (for example Foundation Tany Meva or Madagascar Foundation for Protected Areas and Biodiversity/Madagascar Biodiversity Fund/ Fondation pour les Aires protégées et la biodiversité de Madagascar FAPBM )?
- If yes, how did this funding work compared to traditional donor project funding?

Do you have experiences with funding that has gone to capacity building initiatives (education, training, participation in workshops/conferences?)

If yes, how successful in achieving their objectives were these types of projects compared to supporting other types of activities?

**Challenges and ways forward**

1. What do you think are currently the main challenges for funding biodiversity conservation in Madagascar?
2. Have you seen changes for the better/worse in the funding situation? What and how?
3. Can you think of any improvements in how funding is used to support environmental conservation in Madagascar?
4. Are there particular types of programs, activities, or strategies that have received a) too little or b) too much funding? *(mention only if needed: e.g. marine vs terrestrial; strict PAs vs community managed areas etc.)*

**Finally, just a few questions in relation to the landscape of stakeholders:**

1. Does your organization collaborate with other organizations? local NGOs? international NGOs, state institutions (MNP, others), others? *(if they say yes, then ask who, and in what way they have collaborated)*
2. Is it common that people work for different organizations within the conservation sector over the course of their career; if so, what is the most common scenario of movement? (*if needed, specify for example from local NGOs to state institutions, or from state institutions to international NGOs etc.)*
3. Based on your experiences, how do you see the relationship between local conservation NGOs and international conservation NGOs?

*(only ask if they need help to understand the question: do you see it as positive or are there some difficulties? why or why not?)*

1. Based on your experiences, how do you see the relationship between local conservation NGOs working with each other?

*(only ask if they need help to understand the question: do you see it as positive or are there some difficulties? why or why not?)*

1. Based on your experiences, how do you see the relationship between international conservation NGOs working with each other?

*(only ask if they need help to understand the question: do you see it as positive or are there some difficulties? why or why not?)*

Is there some further information or documentation that might be of interest for us in this project and when planning ahead for the coming research activities?

- We are planning a participatory workshop by the end of the year and happy to hear comments and suggestions of key issues to address?
- Funding data for different PAs, projects etc.?
- Any annual reports that we could gain access to *(ask this only if the previous doesn’t work, here really try to fish for funding data, be persistent and try to go around and get back to it).*
